# Analysis of Correlation in MEMS Gyroscope Array and its Influence on Accuracy Improvement for the Combined Angular Rate Signal

**DOI:** 10.3390/mi9010022

**Published:** 2018-01-09

**Authors:** Liang Xue, Xinguo Wang, Bo Yang, Weizheng Yuan, Guangmin Yuan

**Affiliations:** 1 Xi’an Research Inst. of Hi-Tech, Hongqing Town, Xi’an 710025, China; wxgsea@163.com (X.W.); yangbo8093@sina.com (B.Y.); 2 Ministry of Education Key Laboratory of Micro and Nano Systems for Aerospace, Northwestern Polytechnical University, No. 127 Youyi West Road, Xi’an 710072, China; yuanwz@nwpu.edu.cn (W.Y.); yuangm@nwpu.edu.cn (G.Y.)

**Keywords:** mircoelectromechanical system (MEMS) gyroscope, noise correlation, sensor array, influence analysis, accuracy improvement

## Abstract

Obtaining a correlation factor is a prerequisite for fusing multiple outputs of a mircoelectromechanical system (MEMS) gyroscope array and evaluating accuracy improvement. In this paper, a mathematical statistics method is established to analyze and obtain the practical correlation factor of a MEMS gyroscope array, which solves the problem of determining the Kalman filter (KF) covariance matrix **Q** and fusing the multiple gyroscope signals. The working principle and mathematical model of the sensor array fusion is briefly described, and then an optimal estimate of input rate signal is achieved by using of a steady-state KF gain in an off-line estimation approach. Both theoretical analysis and simulation show that the negative correlation factor has a favorable influence on accuracy improvement. Additionally, a four-gyro array system composed of four discrete individual gyroscopes was developed to test the correlation factor and its influence on KF accuracy improvement. The result showed that correlation factors have both positive and negative values; in particular, there exist differences for correlation factor between the different units in the array. The test results also indicated that the Angular Random Walk (ARW) of 1.57°/h^0.5^ and bias drift of 224.2°/h for a single gyroscope were reduced to 0.33°/h^0.5^ and 47.8°/h with some negative correlation factors existing in the gyroscope array, making a noise reduction factor of about 4.7, which is higher than that of a uncorrelated four-gyro array. The overall accuracy of the combined angular rate signal can be further improved if the negative correlation factors in the gyroscope array become larger.

## 1. Introduction

Small, cheap and precise have become notable characteristics for the future of navigation and guidance systems. Microelectromechanical system (MEMS) inertial sensors are particularly suitable for constructing a compact and low-cost strap-down inertial navigation system because of their prominent characteristics of cheapness, high reliability, small size and low power consumption [1], and even traditional laser and fiber-optic navigation systems have been gradually replaced by MEMS navigation systems in some fields. The performance of MEMS inertial navigation systems is mainly determined by the precision of a micro inertial measurement unit (MIMU), which is used to measure angular rate and the acceleration of the vehicle.

Several methods have been explored to improve MEMS gyroscope accuracy at a device level [2,3,4]. In recent years, other efforts and extensive research has also been undertaken on the accuracy improvement of gyroscopes. One of the competitive technologies is the photonic resonant micro optical gyroscope (RMOG) [5,6,7,8]. Ciminelli and Dell’Olio et al. reported on large InGaAsP/InP ring resonators for gyroscope applications in [5], where the device configuration includes a ring and a straight bus waveguide with tapered ends. Furthermore, a large-radius InP resonator with a high Q-factor of 10^6^ was designed, fabricated, and characterized for the first time in [6]. In particular, the sensing element of a photonic InP-based gyroscope was designed, fabricated, and optically characterized by Ciminelli et al. [8], in which the sensing element is a spiral resonator coupled to a straight bus waveguide through a multimode interference coupler, and exhibits a Q-factor of approximately 600,000 with a footprint of approximately 10 mm^2^; here, the actual feasibility of a photonic gyro on a chip through an established InP-based generic integration process was demonstrated for the first time. Technology for RMOG is also focusing on accuracy improvement at device level and making further progress for the accuracy of gyroscopes.

Previous studies have highlighted that three single gyroscopes configured on each sensitive axis of a MIMU have been unable to provide an angular rate signal with a low drift error for long-duration navigation, especially in such environments where the signals of satellites and geomagnetic and scene-matching systems are seriously disturbed or lacking. The technology of multiple-sensor fusion provides a new way for improving the precision of a MIMU [9,10,11,12,13,14]. An array of MEMS gyroscopes can be configured and mounted on each orthogonally sensitive axis of a MIMU to provide redundant signals at the same condition. Then, the fusion of multiple outputs of a gyroscope array could improve the precision of angular rate measurement. The combined angular rate signals, together with accelerometer signals, can be used for resolving the navigation parameter. Compared to a laser or fiber-optic inertial measurement unit, such a MIMU system could provide equal or better accuracy by employing some appropriate signal-processing algorithm, in addition to being of lower cost, smaller size and higher reliability.

In the design of such a MIMU system based on gyroscope array fusion, the key is the modeling and processing of multiple angular rate signals on each sensitive axis of the MIMU. In 2003, Bayard and Ploen first proposed virtual gyroscope technology to combine four separate MEMS gyroscopes to reduce noise and improve overall accuracy [15]. The simulated results showed that the performance of individual gyroscopes could be effectively improved while giving a favorable correlation. In addition, a MEMS gyroscope array composed of three individual gyroscopes was presented by Chang et al. [16], in which a two-level Kalman filter (KF) scheme was designed to reduce the gyroscope’s drift and improve the accuracy. In particular, the performance of a KF approach for fusing six fully uncorrelated MEMS gyroscopes was further analyzed and evaluated in [17], and it demonstrated that performance can be better than that of an averaging process. Additionally, Tanenhaus et al. reported a method for constructing a MIMU [9,11] in which multiple gyroscopes are placed on each sensitive axis of the MIMU, and a wavelet de-noising method was used to combine outputs of the gyroscope array. Moreover, Lucian et al. also designed a redundant inertial attitude measurement system by placing four separated gyroscopes on each sensitive axis of a MIMU [18]; it used a weighted statistical method for making signal fusion of multiple gyroscopes through setting a weighted factor associated for each sensor. Additionally, a virtual system consisting of four accelerometers and three gyroscopes mounted at designated positions was designed for improving the measurement accuracy of angular rate in [19]; in particular, angular acceleration was calculated by the outputs of accelerometer array based on the geometry relation of the sensors, and then an optimal estimate of input angular rate was obtained by the KF from the measurements of the gyroscope array and angular acceleration.

Previous research has demonstrated that noise and bias instability that exists in individual gyroscopes could be reduced through a fusion of a gyroscope array. However, a favorable correlation that exists in the gyroscope array is the basis for achieving significant accuracy improvement. Obtaining practical correlation factors is a prerequisite for the fusion of multiple gyroscope signals and the evaluation of system performance; thus the aforementioned MIMU system based on a sensor array could be successful implemented. However, unfortunately, previous studies mostly focus on the gyroscope array model, and few of them analyze the sensor’s correlation; it is difficult to effectively analyze and obtain the correlation factor in a MEMS gyroscope array, thus the system covariance matrix **Q** cannot be exactly determined in the implementation of KF. Furthermore, the influence of correlation on the accuracy of the combined angular rate signal cannot be evaluated.

The correlation of a gyroscope array is referred to as the correlation between the gyroscope units, which can be interpreted as the outputs of the component gyroscopes that satisfy a statistical relationship. This relationship can be characterized and indicated by a correlation factor *ρ* and a correlation matrix, where the non-diagonal elements of the matrix can be determined by a correlation factor. In our previous work [16], we supposed that a constant cross-correlation exists between the rate random walk (RRW) noises of the component gyroscopes in a gyroscope array. We attempted to select a different correlation factor to form the KF covariance matrix **Q** for the fusion of outputs of the array, and then the chosen correlation factor corresponding to the minimum drift error of the combined angular rate signal can be regarded as the practical correlation factor in the gyroscope array. However, due to the fact that the KF performance can be affected by other factors, the correlation factor obtained by the above approach may not accurately reflect the actual noise correlation of the gyroscope array; thus the correlation factor setting in the KF system may not match the statistical distribution of the actual noise in the gyroscope array, and may lead to a distortion of the rate signal estimate. In addition, the MEMS gyroscope noise has a slow time-varying random characteristic, and the noise parameters are sensitive to working conditions such as temperature and operating voltage [20]; in particular, the noise variances may vary with the changing of operating conditions. The random characteristic of MEMS gyroscope noise makes it difficult to obtain the correlation factor by using an exact solution.

Therefore, the focus of this work is mainly on the analysis of correlation for a MEMS gyroscope array and its influence on accuracy improvement. A mathematical statistics method is presented to analyze and obtain the practical correlation factor of a MEMS gyroscope array, which can be used to determine the KF covariance matrix **Q** for successfully fusing multiple signals. Based on our previous research regarding multiple signal fusion of a gyroscope array based on a typical gyroscope noise model, the influence of a correlation factor on the drift error of the combined angular rate signal is analyzed by theoretical analysis and computer simulation. Finally, the practical correlation factor and accuracy improvement are tested and analyzed by a four-gyro array experiment. The objective of this paper is to obtain the correlation factor in a MEMS gyroscope array and analyze the influence of correlation on accuracy improvement. This research will provide a useful approach for selecting specific gyroscope units to form an optimal array, which have favorable correlation factors for achieving maximum improvement.

## 2. Mathematical Model of the Multiple Sensors Fusion

**Figure 1:**
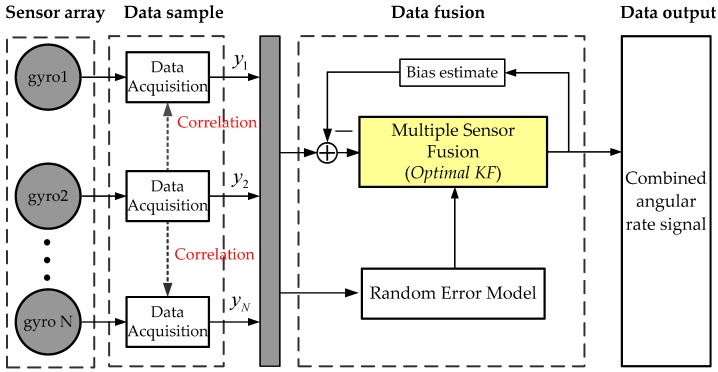
Principle of the multiple rate signals fusion of a MEMS gyroscope array.

The principle of multiple signal fusion of a MEMS gyroscope array is shown in Figure 1, in which the KF technique is used for fusing multiple rate signals to produce an optimal estimate of angular rate signal and drift errors of array. In particular, a correlation between the gyroscope units is identified to reduce noise, and it will affect the final accuracy of the combined angular rate signal. Obtaining a practical correlation factor is very important for the fusion of a gyroscope array. The KF algorithm for combining outputs of a gyroscope array is briefly described as follows.

In previous research [15,17,20], a typical stochastic error model is used to describe the MEMS gyroscope as follows:

(1) $$y(t)=\omega (t)+b(t)+n(t),\;\dot{b}(t)=w_{b}(t)$$

where *y*(*t*) is the output rate signal of gyroscope, *ω*(*t*) is the input true rate signal, *b*(*t*) is the bias drift, driven by a white noise *w_b_* denoted as Rate Random Walk (RRW), and *n*(*t*) is a white noise denoted as Angular Random Walk (ARW).

As for a MEMS gyroscope array with a number of *N*, the gyroscope error model of Equation (1) can be written as a vector form:

(2) $$Z={\left[1,1,\cdots ,1\right]}_{1\times N}^{T}\cdot \omega +b+v,\;\dot{b}=w_{b}$$

with:

$$Z={\left[y_{1},y_{2},\cdots ,y_{N}\right]}^{T},b={\left[b_{1},b_{2},\cdots ,b_{N}\right]}^{T},w_{b}={\left[w_{b1},w_{b2},\cdots ,w_{bN}\right]}^{T},v={\left[n_{1},n_{2},\cdots ,n_{N}\right]}^{T}$$

where *y*_i_ is the output signal of the *i*th gyroscope, **Z**(*t*) is the outputs of the gyroscope array in one sensitive axis, *b*_i_ is the bias drift of the *i*th gyroscope, *w_b_*_i_ and *n*_i_ is the RRW and ARW noise of the *i*th gyroscope, respectively.

A KF technique is utilized to design an optimal filtering algorithm for estimating bias drift **b** and input true rate signal *ω*, thus the KF state vector is comprised by **X**(*t*) = [**b**, *ω*]*^T^*, where the rate signal *ω* is modeled as a random walk process [15,21]:

(3) $$\dot{\omega }=n_{\omega }$$

with *E*[*n_ω_*(*t*)] = 0 and *E*[*n_ω_*(*t*)*·n_ω_^T^*(*t + τ*)] *= q_ω_δ*(*τ*), and *q_ω_* is the variance of white noise *n_ω_*. Based on Equations (2) and (3), the filtering state-space model for combining multiple MEMS gyroscopes can be formed as [22]:

(4) $$\left\{ \begin{array}{l} X(t)={\left[b,\;\omega \right]}^{T}\\ \dot{X}(t)=F\cdot X(t)+w(t)\\ Z(t)=H\cdot X(t)+v(t) \end{array}\right.$$

where **w**(*t*) = [**w***_b_*,*n_ω_*]*^T^* is the system process noise and **v**(*t*) is the measurement noise, which both of them are white noise with the variance as:

(5) $$\left\{ \begin{array}{ll} E\left[w(t)\right]=0, & E\left[w(t)w^{T}(t+\tau )\right]=Q\delta (\tau )\\ E\left[v(t)\right]=0, & E[v(t)v^{T}(t+\tau )]=R\delta (\tau )\\ E\left[w_{b}(t)\right]=0, & E[w_{b}(t)w_{b}{}^{T}(t+\tau )]=Q_{b}\delta (\tau ) \end{array}\right.,Q=\left[\begin{array}{l} Q_{b}\;0\\ 0\;q_{\omega } \end{array}\right]$$

The KF coefficient matrices **F** and **H** can be referred to in [17]. If we suppose that correlation exists in the sensor array between the RRW noises of the component gyroscopes, the correlated covariance matrix **Q***_b_* in Equation (5) can be determined by the correlation factor and RRW noise variance in an off-diagonal form as:

(6) $$Q_{b}={\left[\begin{array}{cccc} \sigma_{b1}^{2} & \rho_{12}\cdot \sqrt{\sigma_{b1}^{2}\sigma_{b2}^{2}} & \cdots & \rho_{1N}\cdot \sqrt{\sigma_{b1}^{2}\sigma_{bN}^{2}}\\ \rho_{21}\cdot \sqrt{\sigma_{b2}^{2}\sigma_{b1}^{2}} & \sigma_{b2}^{2} & \cdots & \rho_{2N}\cdot \sqrt{\sigma_{b2}^{2}\sigma_{bN}^{2}}\\ \vdots & \vdots & \ddots & \vdots \\ \rho_{N1}\cdot \sqrt{\sigma_{bN}^{2}\sigma_{b1}^{2}} & \rho_{N2}\cdot \sqrt{\sigma_{bN}^{2}\sigma_{b2}^{2}} & \cdots & \sigma_{bN}^{2} \end{array}\right]}_{N\times N}$$

where $\sigma_{b,i}^{2}$ is the variance for RRW of the *i*th gyroscope, and *ρ_ij_* is the correlation factor between the *i*th and *j*th gyroscopes of the array corresponding to the RRW noise (*i* = 1,2,…,*N*, *j* = 1,2,…,*N*). Thus, in order to exactly determine the covariance matrix **Q***_b_* and **Q**, the correlation factor *ρ_ij_* should be obtained beforehand.

Through making a discretization of the continuous-time KF state-space model of Equation (4), a discrete iterative KF approach described by Equations (7)–(10) are used to implement KF, and then an optimal estimate of the state vector **X**(*t*) composed of bias drifts **b** and input angular rate signal *ω* could be obtained by the following discrete iterative equations.

(7) $$P_{k/k-1}=F_{k,k-1}P_{k-1}F_{k,k-1}^{T}+Q_{k-1}$$

(8) $$K_{k}=P_{k/k-1}H_{k}^{T}{\left(H_{k}P_{k/k-1}H_{k}^{T}+R_{k}\right)}^{-1}$$

(9) $$P_{k}=(I-K_{k}H_{k})P_{k/k-1}{\left(I-K_{k}H_{k}\right)}^{T}+K_{k}R_{k}K_{k}^{T}$$

(10) $${\hat{X}}_{k}=F_{k,k-1}{\hat{X}}_{k-1}+K_{k}(Z_{k}-H_{k}F_{k,k-1}{\hat{X}}_{k-1})$$

**Figure 2:**
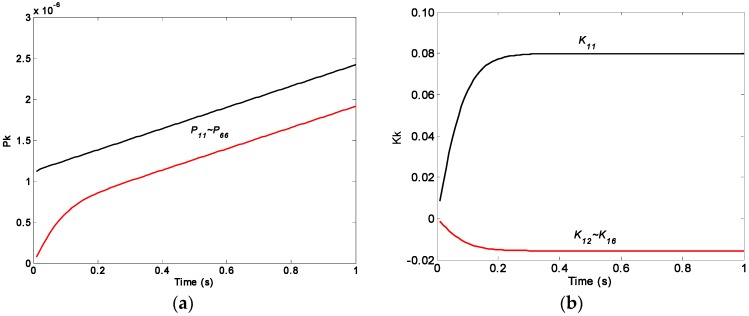
Plot of the KF covariance **P**(*t*) and gain **K**(*t*): (**a**) covariance **P**(*t*); (**b**) gain **K**(*t*).

In order to discover the inherent property of KF, the feature of covariance **P**(*t*) and gain **K**(*t*) are off-line analyzed by Equations (7)–(9), and the plots are shown in Figure 2, where the ARW and RRW noise for the component gyroscopes are set as 0.1667°/h^0.5^ and 600°/h^1.5^, respectively. The period of KF operation and iterative step are set as 0.01 s and 100.

The plot of Figure 2 illustrates that the component values of the matrix **P**(*t*) will be linearly increased with increasing iteration time, and will be diverged without approaching a steady-state value, but the component values of the matrix **K**(*t*) approaches a steady-state value. In addition, the steady and convergent property of the gain **K**(*t*) cannot be influenced by changing the filtering parameters. Thus, in this work, an optimal estimate of input rate signal *ω* could be achieved by using a steady-state gain **K*_S_***, which is obtained by an off-line estimation approach, resulting in a simplified implementation of KF and reduced computational load.

## 3. Analysis of Correlation Factor in MEMS Gyroscope Array

From Section 2, it can be seen that the obtaining of a practical correlation factor *ρ* in a gyroscope array is a crucial aspect for implementation of the KF system, and as a result, the influence of correlation on the accuracy improvement could be further evaluated. In this section, a mathematical statistics method will be established to analyze the correlation of a MEMS gyroscope array and the obtaining of a practical correlation factor.

**Figure 3:**
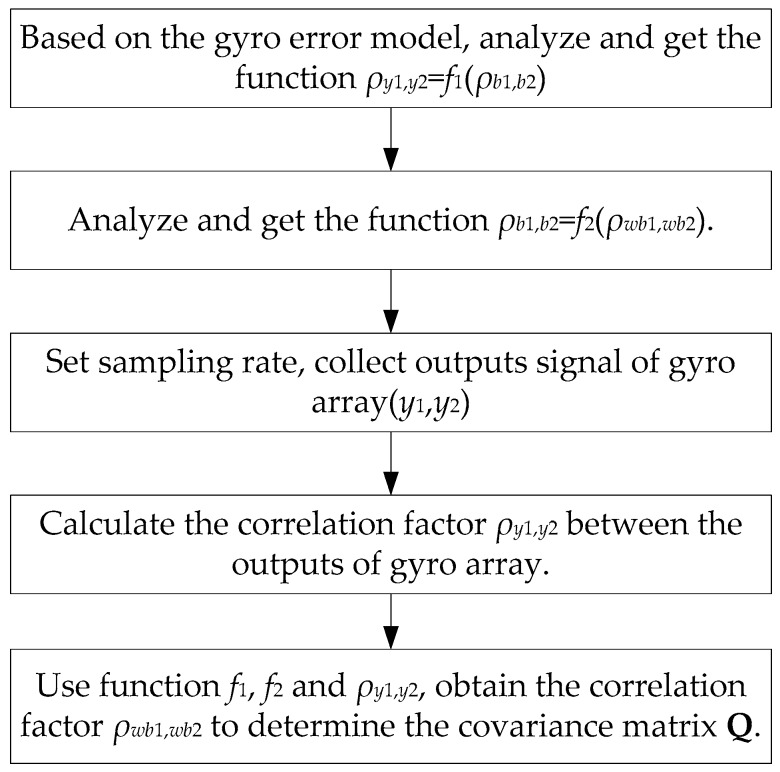
Principle and process flow of analyzing the correlation factor.

In this work, the correlation factor is referred to as the correlation between the same noise items of the gyroscope units. From Equations (1) and (6), the correlation factor *ρ_ij_* represents the correlation between the RRW noises *w_b__i_*(*t*) and *w_b__j_*(*t*), which correspond to the *i*th and *j*th gyroscope in the array, meaning *ρ_ij_* = *ρ_wb__i_*_,*wb*_*_j_*. In the following, two gyroscope units are selected as an example to analyze the relationship between correlation factor *ρ_y_*_1,*y*2_ of *y*_1_ with *y*_2_ and *ρ_wb_*_1,*wb*2_ of RRW *w_b_*_1_(*t*) with *w_b_*_2_(*t*). Based on this relationship, the correlation factor *ρ_wb_*_1,*wb*2_ can be obtained, in that *ρ_y_*_1,*y*2_ can be directly calculated from the outputs of the gyroscope array. The principle and process flow of analyzing the correlation factor is shown in Figure 3. It can be mainly divided into three steps:

- **Step 1:** Analyze the relationship between the correlation factor *ρ_y_*_1,*y*2_ and *ρ_b_*_1,*b*2_ of bias drift *b*_1_ with *b*_2_, get the function *ρ_y_*_1,*y*2_ = *f*_1_(*ρ_b_*_1,*b*2_).
- **Step 2:** Analyze the relationship between the correlation factor *ρ_b_*_1,*b*2_ and *ρ_wb_*_1,*wb*2_ of RRW *w_b_*_1_(*t*) with *w_b_*_2_(*t*), get the function *ρ_b_*_1,*b*2_ = *f*_2_(*ρ_wb_*_1,*wb*2_).
- **Step 3:** Use the functions *f*_1_, *f*_2_ and *ρ_y_*_1,*y*2_ to obtain the correlation factor *ρ_wb_*_1,*wb*2_ to form the covariance matrix **Q***_b_* and **Q**.

➢ **Step 1:**

Under the same conditions, assume that the outputs of the two gyroscope units including an identical input true angular rate and different random noise are sampled and obtained as:

(11) $$\left\{ \begin{array}{l} y_{1}(m)=\omega (m)+b_{1}(m)+n_{1}(m)\\ y_{2}(m)=\omega (m)+b_{2}(m)+n_{2}(m) \end{array}\right.$$

From the definition of cross-correlation function, its value between the sequences of *y*_1_(*m*) and *y*_2_(*m*) at *τ* = 0 can be formed as:

(12) $${R_{y_{1},y_{2}}(\tau )|}_{\tau =0}=R_{y_{1},y_{2}}(0)=\underset{M\to \infty }{\lim}\frac{1}{M}\sum_{m=0}^{M-1}\left[y_{1}(m)\cdot y_{2}(m)\right]$$

As for a gyroscope array with the same specification for component gyroscopes, the sequences of *b*_1_ and *n*_2_ as well as *b*_2_ and *n*_1_ can be considered to be independent. It is essential to assume that the sequences of *n*_1_ and *n*_2_ are independent when analyzing the relationship between the correlation factors *ρ_y_*_1,*y*2_ and *ρ_b_*_1,*b*2_. With the condition of a static test, inserting Equation (11) into (12) yields:

(13) $$R_{y_{1},y_{2}}(0)=R_{b_{1},b_{2}}(0)$$

From Equation (1), it can be seen that the mean values of the sequences *n* and *b* both are zero and independent with each other:

(14) $$\left\{ \begin{array}{l} \mu_{b_{i}}=\frac{1}{M}\sum_{m=0}^{M-1}b_{i}(m)=0,\;i=1,2\\ \mu_{n_{i}}=\frac{1}{M}\sum_{m=0}^{M-1}n_{i}(m)=0,\;i=1,2\\ \mu_{b_{i}}\mu_{n_{i}}=\frac{1}{M}\sum_{m=0}^{M-1}\left[b_{i}(m)\cdot n_{i}(m)\right]=0,\;i=1,2 \end{array}\right.$$

Using Equation (14), the variance of sequences *y*_1_(*m*) and *y*_2_(*m*) are given as:

(15) $$\sigma_{y_{1}}^{2}=\frac{1}{M}\sum_{m=0}^{M-1}{\left[y_{1}(m)-\mu_{y_{1}}\right]}^{2}=\frac{1}{M}\sum_{m=0}^{M-1}{\left[b_{1}(m)\right]}^{2}+\frac{1}{M}\sum_{m=0}^{M-1}{\left[n_{1}(m)\right]}^{2}=\sigma_{d_{1}}^{2}+\sigma_{n_{1}}^{2}$$

(16) $$\sigma_{y_{2}}^{2}=\frac{1}{M}\sum_{m=0}^{M-1}{\left[y_{2}(m)-\mu_{y_{2}}\right]}^{2}=\frac{1}{M}\sum_{m=0}^{M-1}{\left[b_{2}(m)\right]}^{2}+\frac{1}{M}\sum_{m=0}^{M-1}{\left[n_{2}(m)\right]}^{2}=\sigma_{d_{2}}^{2}+\sigma_{n_{2}}^{2}$$

where $\sigma_{n}^{2}$ and $\sigma_{d}^{2}$ are the variance of the sequences *n* and *b*, respectively. From the definition of correlation factor, and the use of Equations (13), (15) and (16), it can be obtained:

(17) $$\rho_{y_{1},y_{2}}=\frac{\rho_{b_{1},b_{2}}}{\sqrt{\left(\sigma_{d_{1}}^{2}+\sigma_{n_{1}}^{2})(\sigma_{d_{2}}^{2}+\sigma_{n_{2}}^{2}\right)}/\sigma_{d_{1}}\sigma_{d_{2}}}$$

where *ρ_b_*_1,*b*2_ is the correlation factor between the bias drift *b*_1_ and *b*_2_ in the gyroscope array, and *ρ_y_*_1,*y*2_ is the correlation factor between the gyroscope outputs of *y*_1_ and *y*_2_. The variances of $\sigma_{n}^{2}$ and $\sigma_{d}^{2}$ can be obtained by the Allan variance technique [23]. Therefore, under the condition of static test, by the use of Equation (17), the correlation factor *ρ_b_*_1,*b*2_ can be obtained from the *ρ_y_*_1,*y*2_ directly computed from the outputs of the gyroscope units.

➢ **Step 2:**

The value of cross-correlation function between the sequences of *b*_1_ and *b*_2_ at *τ* = 0 is formed as:

(18) $$R_{b_{1},b_{2}}(0)=\underset{M\to \infty }{\lim}\frac{1}{M}\sum_{m=0}^{M-1}\left[b_{1}(m)\cdot b_{2}(m)\right]$$

From the discrete form of the random walk model, it yields:

(19) $$\left\{ \begin{array}{l} b_{1,m}=b_{1,m-1}+w_{b1,m-1}\\ b_{2,m}=b_{2,m-1}+w_{b2,m-1} \end{array}\right.$$

And then results in:

(20) $$\begin{array}{ll} (1). & b_{1,m}\cdot b_{2,m}=\underset{↙}{\underset{︸}{b_{1,m-1}b_{2,m-1}}}+b_{1,m-1}w_{b2,m-1}+b_{2,m-1}w_{b1,m-1}+w_{b1,m-1}w_{b2,m-1}\\ (2). & b_{1,m-1}b_{2,m-1}=\underset{↙}{\underset{︸}{b_{1,m-2}b_{2,m-2}}}+b_{1,m-2}w_{b2,m-2}+b_{2,m-2}w_{b1,m-2}+w_{b1,m-2}w_{b2,m-2}\\ (3). & b_{1,m-2}b_{2,m-2}=\underset{↙}{\underset{︸}{b_{1,m-3}b_{2,m-3}}}+b_{1,m-3}w_{b2,m-3}+b_{2,m-3}w_{b1,m-3}+w_{b1,m-3}w_{b2,m-3}\\ (4). & b_{1,m-3}b_{2,m-3}=\underset{↙}{\underset{︸}{b_{1,m-4}b_{2,m-4}}}+b_{1,m-4}w_{b2,m-4}+b_{2,m-4}w_{b1,m-4}+w_{b1,m-4}w_{b2,m-4}\\ \;\vdots & \;\vdots \\ (m). & \;b_{1,1}b_{2,1}=b_{1,0}b_{2,0}+b_{1,0}w_{b2,0}+b_{2,0}w_{b1,0}+w_{b1,0}w_{b2,0} \end{array}$$

Using Equation (20), Equation (18) can be expressed as:

(21) $$R_{b_{1},b_{2}}(0)=\underset{M\to \infty }{\lim}\frac{1}{M}\sum_{m=0}^{M-1}\left[b_{1}(m)\cdot b_{2}(m)\right]=\underset{M\to \infty }{\lim}\frac{1}{M}\sum_{m=1}^{M-1}\left[\sum_{i=0}^{m-1}\left(w_{b_{1},i}w_{b_{2},i}+b_{2,i}w_{b_{1},i}+b_{1,i}w_{b_{2},i}\right)\right]$$

Analysis result of the cross-correlation function indicates that the sequences *b*_1_ and *w_b_*_2_ are independent, and the sequences *b*_2_ and *w_b_*_1_ are also independent. Therefore, the cross-correlation function *R_b_*_1,*b*2_(0) can be expressed as:

(22) $$R_{b_{1},b_{2}}(0)=\underset{M\to \infty }{\lim}\frac{1}{M}\sum_{m=1}^{M-1}\left(w_{b_{1},0}w_{b_{2},0}+w_{b_{1},1}w_{b_{2},1}+\cdots +w_{b_{1},m-1}w_{b_{2},m-1}\right)=M\cdot R_{w_{b1},w_{b2}}(0)$$

Based on the definition of correlation factor and Equation (22), the relationship between correlation factor *ρ_b_*_1,*b*2_ and correlation factor *ρ_wb_*_1,*wb*2_ can be obtained as:

(23) $$\rho_{b_{1},b_{2}}=\frac{R_{b_{1},b_{2}}(0)}{\sigma_{d_{1}}\sigma_{d_{2}}}=M\cdot \frac{R_{w_{b1},w_{b2}}(0)}{\sigma_{d_{1}}\sigma_{d_{2}}}$$

And then:

(24) $$\rho_{b_{1},b_{2}}=M\cdot \frac{\rho_{w_{b1},w_{b2}}}{\sigma_{d_{1}}\sigma_{d_{2}}/(\sigma_{b_{1}}\sigma_{b_{2}})}$$

where $\sigma_{b}^{2}$ is the variance of the sequences of RRW *w_b_*, which can be obtained by the Allan variance technique, and *ρ_wb_*_1,*wb*2_ is the correlation factor between the RRW noise *w_b_*_1_ and *w_b_*_2_ in the gyroscope array. Therefore, in a practical KF implementation, the *ρ_wb__i_*_,*wb*_*_j_* can be calculated by using Equations (17) and (24), and then to determine the covariance matrix **Q** in Equation (5).

## 4. Theoretical Analysis of the Correlation Influence on Noise Reduction

The noise characteristic of the combined angular rate signal and performance of the KF can be analyzed and evaluated by the covariance **P**(*t*).The drift error of the combined angular rate signal can be given as [15]:

(25) $$D_{vg}=\frac{1}{H_{1}{}^{T}Q_{b}^{-1}H_{1}+1/q_{\omega }}$$

The above parameter *q_ω_* reflects the dynamic characteristic of input rate signal. With regard to the KF system, it determines the KF bandwidth. When setting the parameter *q_ω_* as a larger value, the KF bandwidth will become much larger than that of the component gyroscopes [17], and then the system bandwidth is only determined by the component gyroscopes, which will be close to the bandwidth of the component gyroscopes. In this case, the drift of combined angular rate signal is mainly influenced by the correlation factor and number of array *N*, making it easy to analyze the effect of correlation on noise reduction. Therefore, with regard to a gyroscope array with the same specification, assuming that a constant cross-correlation exists in the array, the covariance matrix **Q***_b_* of Equation (6) can be expressed as:

(26) $$Q_{b}=\sigma_{b}^{2}\cdot {\left[\begin{array}{cccc} 1 & \rho & \cdots & \rho \\ \rho & 1 & \cdots & \rho \\ \vdots & \vdots & \ddots & \vdots \\ \rho & \rho & \cdots & 1 \end{array}\right]}_{N\times N}$$

Inserting Equation (26) and substituting *q_ω_* → ∞ into Equation (25) yields:

(27) $$D_{vg}=\sigma_{b}^{2}\frac{1+(N-1)\rho }{N}$$

**Figure 4:**
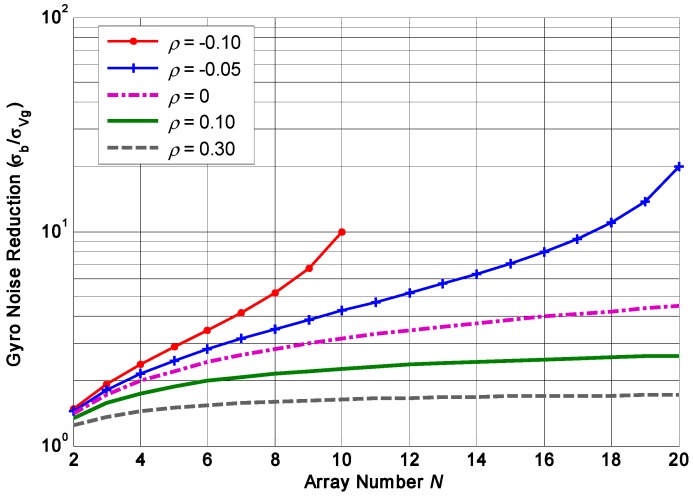
Plot of the relationship between gyroscope drift reduction and array number *N* with various correlation factor (*ρ* = −0.05, −0.1, 0, 0.1, 0.3).

Previous study has demonstrated that a negative correlation factor is much more favorable for achieving higher accuracy improvement. Taking the constant correlation factor *ρ* = −0.1, −0.05, 0, 0.1, 0.3, the plot of gyroscope drift reduction versus number *N* is illustrated in Figure 4. It suggests that the drift reduction ratio will be increased with the increase of number *N*; however, the graph slope gradually becomes shallower with the increase of the number *N* while giving a positive correlation factor (*ρ* = 0.1, 0.3). This implies that the increasing magnitude of the noise reduction is smaller than that of the number *N*, thus the influence of number *N* on accuracy improvement will become smaller and more insignificant as *N* increases. On the contrary, giving a negative correlation factor (*ρ* = −0.1, −0.05), the graph slope steepens with the increase of the number *N*, which suggests that the influence of increasing number *N* on accuracy improvement will be much more evident and significant. Consequently, the number *N* needs to increase appropriately to obtain a higher improvement when the gyroscope array has a negative correlation. In Section 5.1, the influence of the constant cross-correlation on the drift of the combined angular rate signal will be analyzed in various simulations.

## 5. Simulation and Experiment

In this section, the influence of the correlation factor on the drift reduction of the combined angular rate signal will be analyzed by simulation and experiment. The noise statistic values of the rate signal before and after KF combining are used as a criterion to evaluate the improvement.

### 5.1. Simulation Result

From the Section 4 it is known that the drift error of the combined angular rate signal will decrease with increase of *N*; the number of *N* = 8 is chosen to implement the system. Assuming that a constant correlation factor exists in the gyroscope array, here, four different correlation factors of *ρ* = {0.2, 0, −0.13, −0.142} are selected to carry out the simulation. The outputs of gyroscope array are generated by model (1) with a sampling period of 0.1 s. The procedure for simulating the outputs of gyroscope array with a specific constant correlation can be summarized as follows:

- Step 1: Use a constant cross-correlation factor *ρ* to form the correlated matrix **CorrM**, which can be referred to from Equation (26), and the **CorrM** should be defined as a positive definite matrix;
- Step 2: Perform the *Cholesky* factorization of matrix **CorrM**, Δ=Chol(CoorM), where Δ is an upper triangular matrix;
- Step 3: Generate the RRW noise data *S_Ind_* of a gyroscope array, and then form the correlated RRW noise data *S_Corr_* with the correlation factor *ρ*, $S_{Corr}=\Delta \cdot S_{Ind}$;
- Step 4: Use the error model (1), generate the output signals of gyroscope array *y*_1_, *y*_2_, …, *y_N_*.

**Figure 5:**
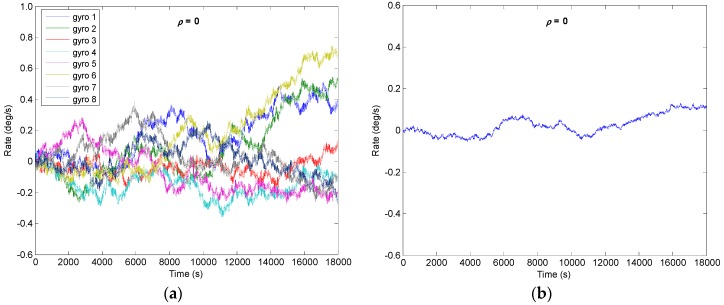
Filtering result of gyroscope array with *ρ* = 0: (**a**) Outputs of component gyroscopes; (**b**) Output of combined gyroscope.

**Figure 6:**
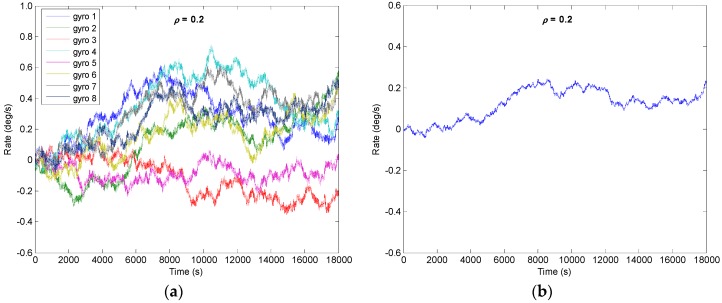
Filtering result of gyroscope array with *ρ* = 0.2: (**a**) Outputs of component gyroscopes; (**b**) Output of combined gyroscope.

**Figure 7:**
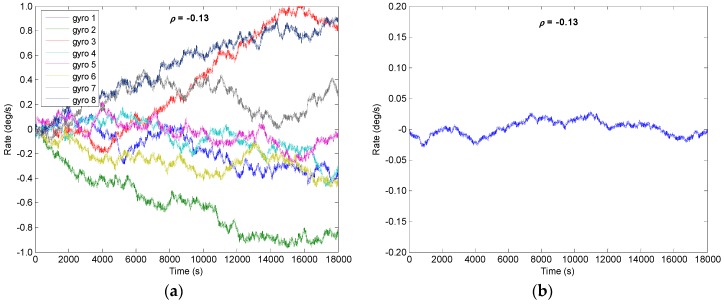
Filtering result of gyroscope array with *ρ* = −0.130: (**a**) Outputs of component gyroscopes; (**b**) Output of combined gyroscope.

**Figure 8:**
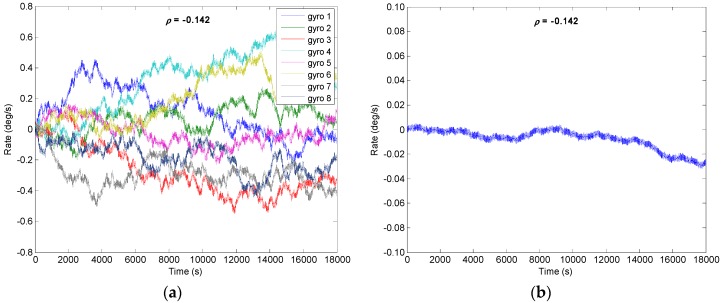
Filtering result of gyroscope array with *ρ* = −0.142: (**a**) Outputs of component gyroscopes; (**b**) Output of combined gyroscope.

**Figure 9:**
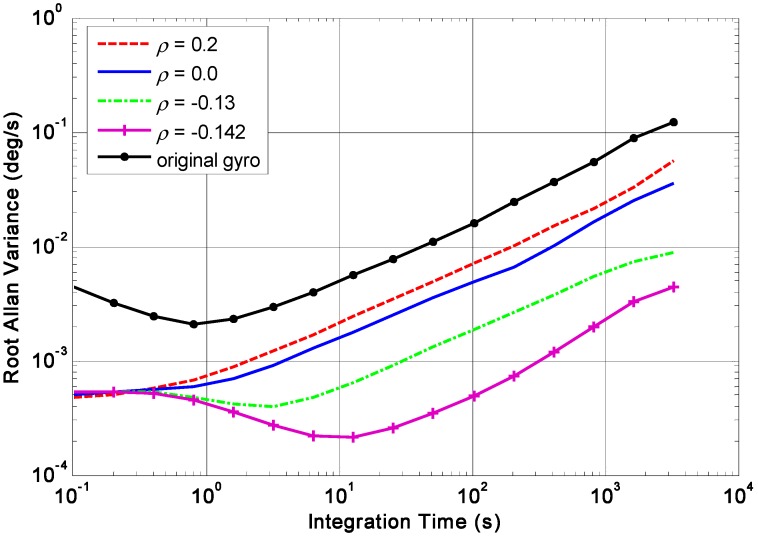
Plot of the compared Allan variance results with different correlation factors (*ρ* = −0.142, −0.13, 0, 0.2).

The RRW and ARW for the component gyroscopes are set as *σ_b_* = 600°/h^1.5^ and *σ_n_* = 0.0833°/h^0.5^, respectively. Using KF discrete recursive equation of (7)–(10) and a steady-state gain **K*_S_***, the results of combined angular rate signal with different correlation factors are shown in Figure 5, Figure 6, Figure 7 and Figure 8, and the plot of compared Allan variance is illustrated in Figure 9. The results are listed in Table 1, where the reduction factor RF associated with RRW noise is defined as:

(28) $$RF=RRW_{single}/RRW_{Vg}$$

where *RF* is the reduction factor, *RRW_single_* is the RRW for the gyroscopes before KF filtering, and *RRW_Vg_* is the RRW for the combined angular rate signal after KF filtering.

The results indicate that the RRW noise for the single gyroscope is remarkably reduced by fusing the multiple outputs of a gyroscope array. Furthermore, the noise reduction factor obtained by a negative correlation is greater than that of a positive one. Especially, the RRW noise of 600°/h^1.5^ is reduced to about 18.6°/h^1.5^ with the correlation factor *ρ* = −0.142, making a reduction factor of about 32. In addition, the gyroscope bias drift has also been reduced.

### 5.2. Experiment Result

In the simulation section, the outputs of a gyroscope array with a specific constant correlation can be intentionally generated to analyze KF performance. However, to date, a practical MEMS gyroscope array with some specific correlation factors has been difficult to intentionally design, thus in the experiment, four individual gyroscopes of *N* = 4 are used to form a gyroscope array that is not consistent with the number *N* = 8 in simulation; it is selected just to test the influence of correlation on accuracy improvement.

**Figure 10:**
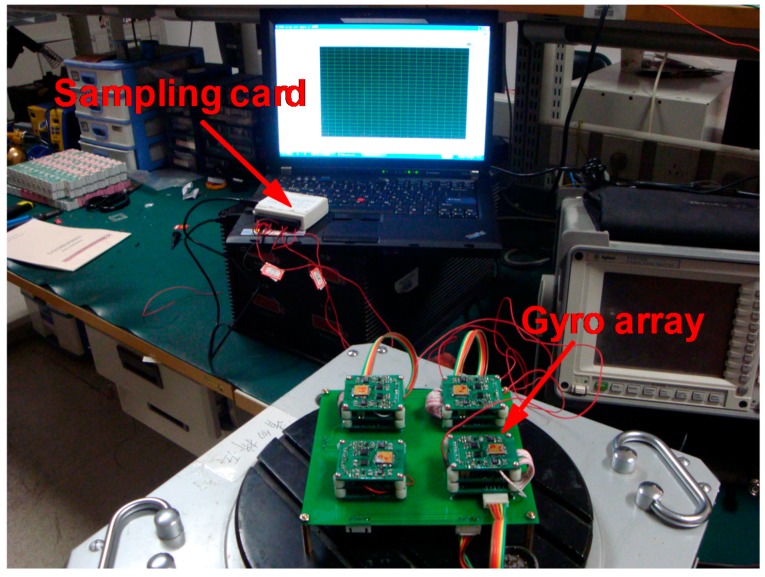
Prototype of the combined system with four-gyro array.

The prototype of the four-gyro array is shown in Figure 10. Firstly, the correlation factors of the four-gyro array are tested by using the approach given in Section 3. Secondly, the bias drift of the combined angular rate signal is tested and compared with the individual gyroscopes.

#### 5.2.1. Correlation Factor in Four-Gyro Array

The correlation factors between the component gyroscopes are not intentionally set in the experiment, thus the practical correlation factors should first be tested and obtained to write them into the KF covariance matrix **Q** (see Equation (6)), and then the outputs of gyroscope array can be processed by KF to achieve a combined angular rate signal. Particularly, the tested correlation factors can be used to analyze the accuracy improvement in theory, which can be compared with the test results.

The cross-correlation matrix is utilized to characterize the RRW noise correlation in the array. Using the approach given in Section 3, the three tests results of cross-correlation matrix are listed in Table 2, Table 3 and Table 4.

The result demonstrates that the correlation factors have both positive and negative values. The maximum positive and negative value is about 0.28 and −0.4, respectively. In addition, there exist differences in the correlation factors between the different gyroscope units, but between the same gyroscope units it is basically consistent, e.g., as for the gyroscope units of 1–2, the correlation factors are 0.27, 0.28 and 0.26 in three tests, while the values are −0.41, −0.40 and −0.43 for units of 1–3. In particular, Table 2, Table 3 and Table 4 illustrate the correlation factors between the units 1–2, 1–3, 2–3 and 3–4 are close to or greater than 0.1, which can be regarded as correlated. By contrast, the values between the units 1–4 and 2–4 are smaller than 0.05, so can be considered to be uncorrelated. Finally, the correlation factors in Table 2, Table 3 and Table 4 can be used to form the covariance matrix **Q***_b_* of Equation (6) to implement KF.

#### 5.2.2. Drift Test Result of the Fused Sensor Array

**Figure 11:**
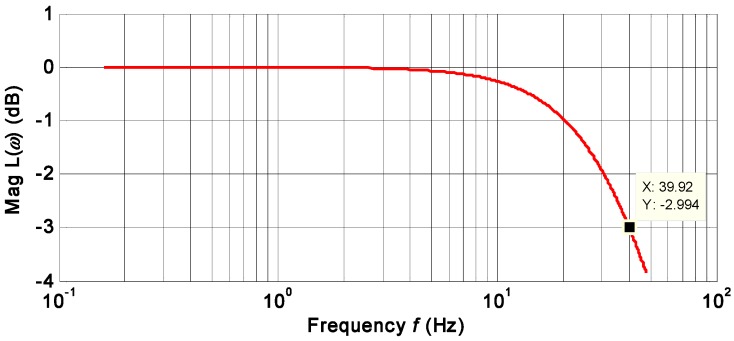
Plot of the frequency response of KF for $\sqrt{q_{\omega }}=11,500{}^{\circ}/h$.

The KF bandwidth is set as the same as the individual gyroscope at 40 Hz to test influence of correlation factor on drift of the combined angular rate signal. From the plot of the KF frequency response (Figure 11), it indicates a −3 dB bandwidth of 40 Hz when choosing the value of $\sqrt{q_{\omega }}=11,500{}^{\circ}/h$.

**Figure 12:**
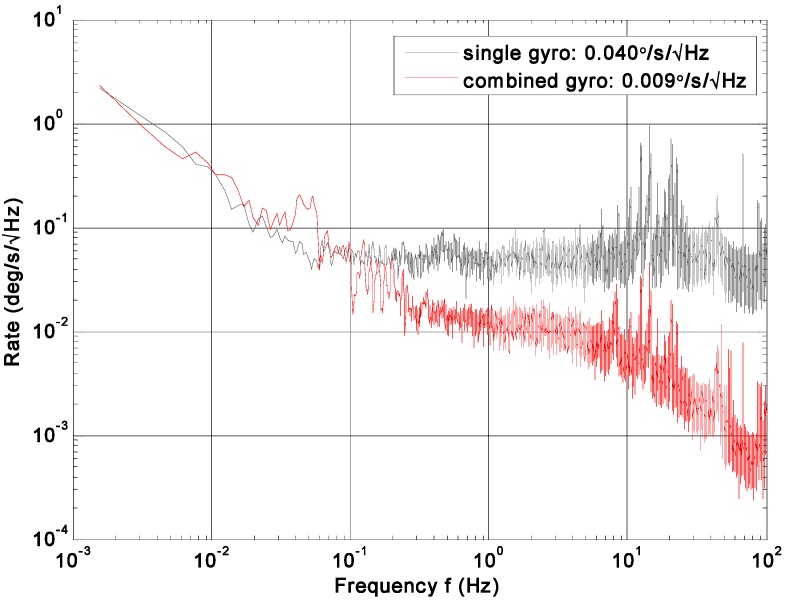
FFT plot of combined angular rate signal compared to the single gyroscope.

**Figure 13:**
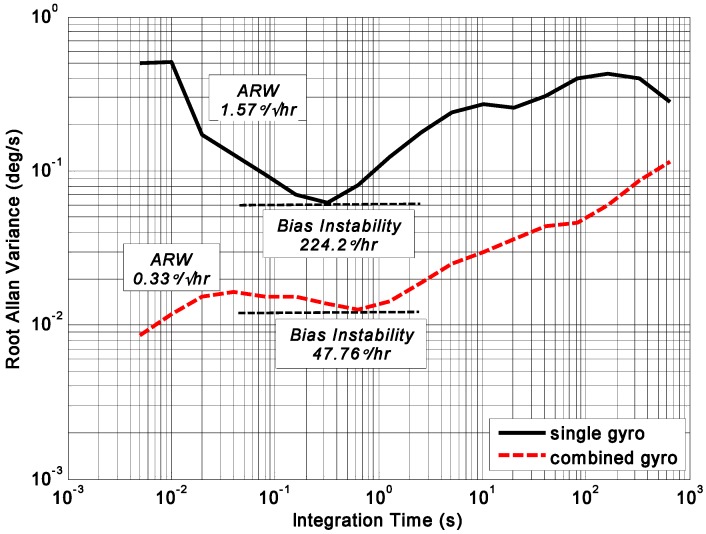
Allan variance results of combined angular rate signal compared to single gyroscope.

The noise density, ARW, and bias drift were tested to evaluate the influence of correlation factor on accuracy improvement. Fast Fourier Transform (FFT) was adopted to evaluate the noise density of rate signal. ARW and bias drift were evaluated by the Allan variance of a zero rate output recorded for 0.5 h with a rate of 200 Hz. The compared plots of noise density and root Allan variance are shown in Figure 12 and Figure 13. The results are listed in Table 5.

The FFT plot indicates a noise floor of about 0.009°/s/Hz^0.5^ and 0.04°/s/Hz^0.5^ for the combined angular rate signal and individual gyroscope, respectively, making a reduction factor of about 4.4. In addition, from Figure 13, ARW and bias drift are observed to be 0.33°/h^0.5^ and 47.8°/h for the combined angular rate signal, which makes a noise reduction factor of about 4.7 compared to the individual gyroscope.

The test results demonstrate that the noise reduction factor is nearly 4.7 for a four-gyro array, having some negative correlation factors (see Table 2, Table 3 and Table 4), while the reduction factor for a completely uncorrelated four-gyro array is only 2.0. It can be seen that negative correlation has a critical effect on accuracy improvement. The overall accuracy can be further improved if the negative correlation factors between the gyroscope units become larger. Additionally, a better consistency of the correlation factors between gyroscope array units is more favorable for improving accuracy. The experimental results are not compared with the simulation, because: (1) the correlation factors between gyroscope units are not consistent in the experiment test, but in the simulation we assume that a constant cross-correlation between gyroscope units exists; (2) the correlation factors in the four-gyro array both have positive and negative values, which is different from the correlation factors in the simulation. In addition, in this study, a reduction factor is used as a criterion for evaluating the influence of correlation factor on accuracy improvement; thus if the component gyroscopes with a lower drift are chosen to form the array, the combined gyroscope rate signal with a better accuracy will be achieved.

## 6. Conclusions

In this paper, a mathematical statistics method is presented to analyze and obtain the practical correlation factors of a MEMS gyroscope array, which solves the problem of determining a KF covariance matrix **Q** and implementing the fusion of multiple signals. It can be used to select gyroscope units with specific correlation to form an optimal array. Both theory and simulation have shown that a negative correlation has a favorable influence on accuracy improvement. The experiment demonstrated that ARW and bias drift are observed to be 0.33°/h^0.5^ and 47.8°/h for the combined gyroscope for a four-gyro array, which makes a noise reduction factor of about 4.7. With regard to a MEMS gyroscope array composed of several discrete individual sensors, the test results displayed that there exist differences in the correlation factors between the different units in the array. This is mainly due to the separate detection circuit and sensitive structure of the component gyroscopes. The influencing factor on the correlation in gyroscope array needs to be further studied in future work, with the hope that some negative correlations could be intentionally designed that would considerably improve accuracy.

## Figures and Tables

**Table 1:** Filtering results of the gyroscope array with different correlation factor.

Correlation Factor	ARW (°/h^0.5^)	Bias Drift (°/h)	RRW (°/h^1.5^)	Reduction Factor (RF)
*ρ* = 0	0.0117	1.8401	195.2674	3.0727
*ρ* = 0.2	0.0112	1.7349	260.4219	2.3039
*ρ* = −0.130	0.0283	1.4410	68.6507	8.7398
*ρ* = −0.142	0.0301	0.7666	18.6092	32.2421

**Table 2:** Cross-correlation matrix of RRW noise for four-gyro array (Test 1).

Gyro Number	Gyro-1	Gyro-2	Gyro-3	Gyro-4
Gyro-1	1	0.2761	−0.4165	−0.0209
Gyro-2	0.2761	1	−0.0800	0.0295
Gyro-3	−0.4165	−0.0800	1	0.0864
Gyro-4	−0.0209	0.0295	0.0864	1

**Table 3:** Cross-correlation matrix of RRW noise for four-gyro array (Test 2).

Gyro Number	Gyro-1	Gyro-2	Gyro-3	Gyro-4
Gyro-1	1	0.2821	−0.4021	−0.0817
Gyro-2	0.2821	1	−0.0919	−0.0612
Gyro-3	−0.4021	−0.0919	1	0.1033
Gyro-4	−0.0817	−0.0612	0.1033	1

**Table 4:** Cross-correlation matrix of RRW noise for four-gyro array (Test 3).

Gyro Number	Gyro-1	Gyro-2	Gyro-3	Gyro-4
Gyro-1	1	0.2638	−0.4271	0.0037
Gyro-2	0.2638	1	−0.0897	0.0229
Gyro-3	−0.4271	−0.0897	1	0.0923
Gyro-4	0.0037	0.0229	0.0923	1

**Table 5:** Drift test results of the four-gyro array.

Terms	Single Gyro	Virtual Gyro	Reduction Factor (RF)
Noise density (°/s/Hz^0.5^)	0.040	0.009	4.44
ARW (°/h^0.5^)	1.57	0.33	4.76
Bias drift (°/h)	224.2	47.76	4.69
